# Enhancement of Anti-Tumoral Immunity by β-Casomorphin-7 Inhibits Cancer Development and Metastasis of Colorectal Cancer

**DOI:** 10.3390/ijms22158232

**Published:** 2021-07-30

**Authors:** Shiori Mori, Rina Fujiwara-Tani, Shingo Kishi, Takamitsu Sasaki, Hitoshi Ohmori, Kei Goto, Chie Nakashima, Yukiko Nishiguchi, Isao Kawahara, Yi Luo, Hiroki Kuniyasu

**Affiliations:** 1 Department of Molecular Pathology, Nara Medical University, 840 Shijo-cho, Kashihara 634-8521, Japan; m.0310.s.h5@gmail.com (S.M.); rina_fuji@naramed-u.ac.jp (R.F.-T.); nmu6429@yahoo.co.jp (S.K.); takamitu@fc4.so-net.ne.jp (T.S.); brahmus73@hotmail.com (H.O.); ilgfgtk@gmail.com (K.G.); c-nakashima@naramed-u.ac.jp (C.N.); yukko10219102@yahoo.co.jp (Y.N.); isao_kawahara@a011.broada.jp (I.K.); 2 Key Laboratory of Neuroregeneration of Jiangsu and Ministry of Education, Co-Innovation Center of Neuroregeneration, Nantong University, Nantong 226001, China

**Keywords:** β-casomorphin-7, lymphocytes, CD8^+^ T cell, colon carcinogenesis, colon cancer metastasis

## Abstract

β-Casomorphin-7 (BCM) is a degradation product of β-casein, a milk component, and has been suggested to affect the immune system. However, its effect on mucosal immunity, especially anti-tumor immunity, in cancer-bearing individuals is not clear. We investigated the effects of BCM on lymphocytes using an in vitro system comprising mouse splenocytes, a mouse colorectal carcinogenesis model, and a mouse orthotopic colorectal cancer model. Treatment of mouse splenocytes with BCM in vitro reduced numbers of cluster of differentiation (CD) 20^+^ B cells, CD4^+^ T cells, and regulatory T cells (Tregs), and increased CD8^+^ T cells. Administration of BCM and the CD10 inhibitor thiorphan (TOP) to mice resulted in similar alterations in the lymphocyte subsets in the spleen and intestinal mucosa. BCM was degraded in a concentration- and time-dependent manner by the neutral endopeptidase CD10, and the formed BCM degradation product did not affect the lymphocyte counts. Furthermore, degradation was completely suppressed by TOP. In the azoxymethane mouse colorectal carcinogenesis model, the incidence of aberrant crypt foci, adenoma, and adenocarcinoma was reduced by co-treatment with BCM and TOP. Furthermore, when CT26 mouse colon cancer cells were inoculated into the cecum of syngeneic BALB/c mice and concurrently treated with BCM and TOP, infiltration of CD8^+^ T cells was promoted, and tumor growth and liver metastasis were suppressed. These results suggest that by suppressing the BCM degradation system, the anti-tumor effect of BCM is enhanced and it can suppress the development and progression of colorectal cancer.

## 1. Introduction

Colorectal cancer is the leading cause of cancer-related death in the world and its incidence has continued to increase in recent years [1]. Many causes of colorectal cancer are lifestyle-related, especially diet. Inflammation caused by fatty acid metabolism and lipid overdose, alcohol drinking, and over-consumption of processed meat are known as risk factors for tumor progression [2].

A correlation between milk intake and reduced risk of colon cancer has been noted [3,4,5]. Milk intake has been reported to reduce the risk of colorectal cancer in men by 25% [6]. In addition, milk intake increases the responsiveness of colorectal cancer to chemotherapy and suppresses metastasis [7]. The involvement of milk components in the anti-tumor effect of milk has been examined. The milk component lactoferrin suppresses colorectal carcinogenesis and metastasis [8]. In contrast, calcium, which is also a milk component, is controversial in terms of colorectal carcinogenesis risk [5,9,10]. Thus, it is not completely clear which milk component has an anti-tumor effect. In the current study, we focused on β-casomorphin-7 (BCM) as a milk component.

BCM is a degradation product of β-casein mainly produced from A1 type in the stomach, and is a peptide consisting of seven amino acids (Tyr-Pro-Phe-Pro-Gly-Pro-Ile) on the C-terminal side. From 1 L of milk, 0.4 g of BCM can be produced [11]. Although several different isoforms are known for BCM, BCM-4, 5, 7, and 8 all have opioid activity, and BCM-7 is the most released from β-casein [12]. BCM is known to be associated with several diseases; β-casein A1 consumption is involved in ischemic heart disease [13]. BCM has also been suggested as a cause of sudden infant death syndrome, with neuropathy such as autism and schizophrenia associated with milk consumption and high levels of BCM [13,14]. In contrast, regarding the association between BCM and cancer, it has been reported that BCM suppresses cell proliferation of prostate cancer and breast cancer via opioid receptors [15]. BCM reduces the expression of tumor necrosis factor-α and phosphorylated inhibitor of nuclear factor κBα/inhibitor of nuclear factor κBα and inactivates nuclear factor κB [16]. Thus, it is suggested that BCM has an anti-tumor effect due to its direct action on cancer cells.

BCM also acts on the immune system. It was found to significantly inhibit DNA synthesis in concanavalin A-stimulated colonic mucosal lymphocytes [17]. Conversely, BCM suppresses lymphocyte proliferation at low concentrations, but promotes it at high concentrations above 10^−7^ M [18] by activating the T cell helper (Th)-2 pathway, which induces an inflammatory response in the gut [19]. Notably, 95% of BCM is degraded by digestive juice and jejunal brush border [20]. Therefore, under such physiological conditions, BCM presents only in low concentrations and might suppress the immune system. In contrast, high concentrations of BCM may provide immunostimulatory properties. This suggests that high concentrations of BCM might promote anti-tumor immunity against colorectal cancer.

In this study, we investigated the effect of BCM on colorectal carcinogenesis and metastasis by inhibiting the BCM degradation system.

## 2. Results

### 2.1. Effect of BCM on Lymphocyte Subsets

**Figure 1:**
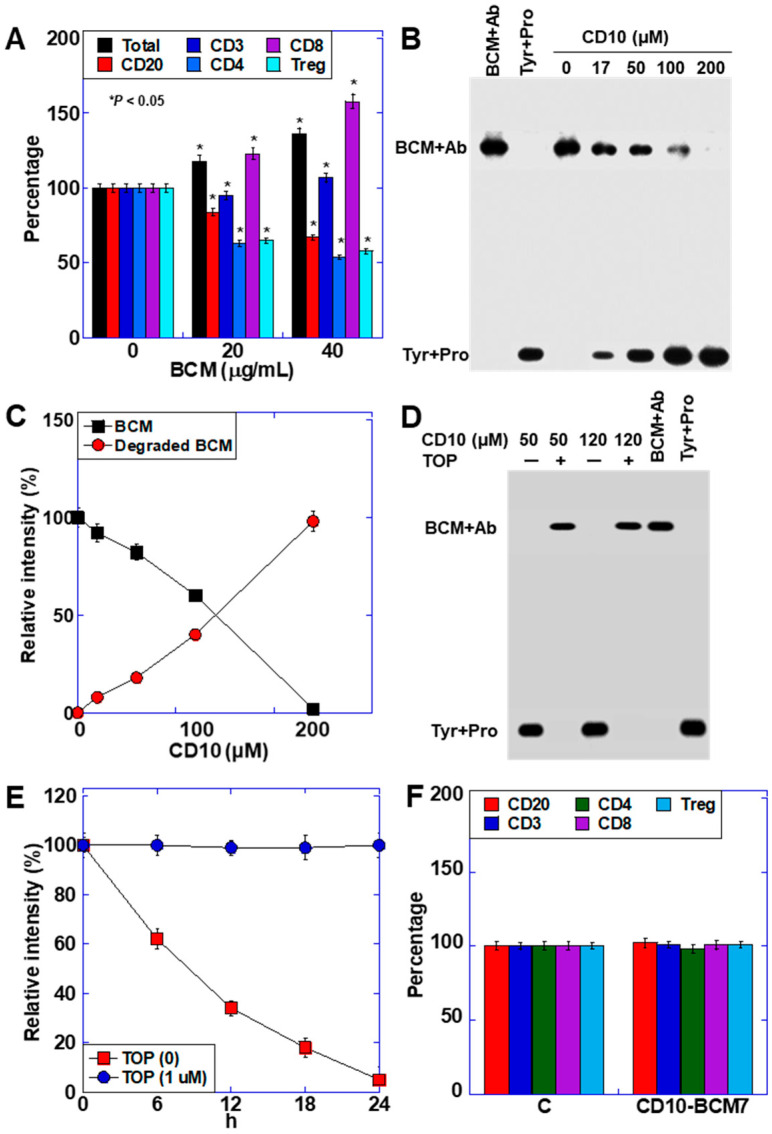
Effect of BCM on spleen cells and degradation of BCM by CD10. (**A**) Effect of BCM on subpopulations of spleen cells. Spleen cells of BALB/c mice were treated with BCM (0.1 μg/mL) [21] for 48 h. Differentiation markers were examined by ELISA. (**B**) Effect of CD10 on degradation of BCM. ^125^I-labeled BCM (50 μg) was incubated with CD10 for 16 h. Intact BCM bound to anti-BCM antibody and degraded peptides were separated with electrophoresis and detected by autoradiography. (**C**) CD10 concentration-dependent degradation of BCM demonstrated by semi-quantification of panel B. (**D**) Effect of TOP on BCM degradation by CD10. ^125^I-labeled BCM (50 μg) was incubated with CD10 and TOP (1 μM). (**E**) Time course of degradation of BCM by CD10. ^125^I-labeled BCM (50 μg) was incubated with CD10 (60 μg) and TOP (1 μM). (**F**) Effect of degraded BCM on subset of spleen cells. Error bar, standard deviation from three independent trials. BCM, β-casomorphin-7; cluster of differentiation; ELISA, enzyme-linked immunosorbent assay; Treg, regulatory T cell; tyr-pro, 1st and 2nd amino acids of BCM; Ab, anti-BCM antibody; TOP, thiorphan.

To examine the effect of BCM on lymphocytes, immune cells collected from the spleen were treated with BCM (Figure 1A). By examining each subset of lymphocytes, we found that the numbers of cluster of differentiation (CD) 8^+^ T cells increased, while that of CD4^+^ T cells and regulatory T cells (Tregs) showed a marked decrease upon BCM treatment. As shown in Table 1, protein levels of phosphorylated phospholipase (pPLC) γ1 (downstream of 5-hydroxytryptamine (HT)-2-serotonin receptor) and histamine were examined. BCM provided downregulation of pPLC in the spleen cells, and an increase of histamine in the culture medium.

### 2.2. Degradation of BCM by CD10

Since neuropeptides such as enkephalin are known to be degraded by CD10, CD10 is also suggested to degrade BCM [20]; as such, we treated BCM with CD10 protein and examined its degradation (Figure 1B,C). The N-terminal tyrosine of BCM was labeled with ^125^I, and then treated with CD10, followed by treatment with an anti-BCM antibody and electrophoresis in a 15–25% polyacrylamide gel. Intact BCM decreased in a CD10 concentration-dependent manner, and at 120 μg/mL of CD10, all intact BCM disappeared in 12 h. Notably, a degraded BCM fragment at a position equivalent to tyr-pro, which are the two amino acids on the N-terminal side of BCM, was observed. It is considered that a short fragment of tyr-pro at the N-terminal of ^125^I-labeled BCM was cleaved. In contrast, when BCM was treated with thiorphan (TOP) (1 μM), an inhibitor of CD10, in combination with CD10, the degradation of BCM was completely suppressed (Figure 1D). A time course analysis of the degradation showed that CD10 at 60 μg/mL decreased intact BCM in a time-dependent manner and intact BCM completely disappeared after 24 h (Figure 1E). In contrast, no decrease in intact BCM was observed when it was additionally treated with TOP. Of note, the CD10 degradation product of BCM had no effect on the lymphocyte subset (Figure 1F).

### 2.3. Effect of BCM on Intestinal Lymphocytes

**Figure 2:**
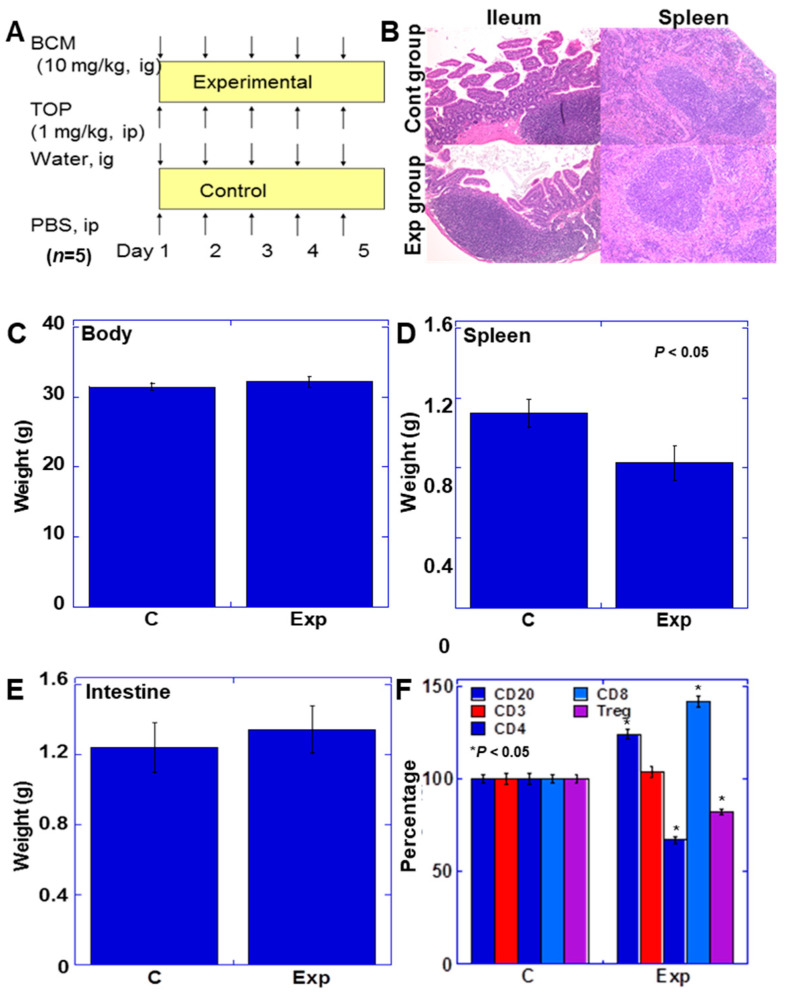
Effect of BCM on lymphatic tissue of mouse spleen and intestine. (**A**) BALB/c mice were treated with BCM and TOP. (**B**) Histological appearance of lymphatic tissues of the ileum and spleen. (**C**–**E**) Weights of the body, spleen, and intestine of BCM+TOP-treated mice. (**F**) Effect of BCM+TOP on lymphocyte subset of the intestinal mucosa. Differentiation markers were examined by ELISA. Error bar, standard deviation from three independent trials. BCM, β-casomorphin-7; cluster of differentiation; TOP, thiorphan; Exp, experimental group; C, control group; ELISA, enzyme-linked immunosorbent assay; Treg, regulatory T cell.

To investigate the effect of BCM on intestinal lymphocytes, we administered BCM and TOP to BALB/c mice and examined changes in the lymphocyte subset (Figure 2). BCM (intragastric gavage) and TOP (intraperitoneal [ip] injection) were administered for five consecutive days, after which the mice were euthanized (Figure 2A). The administration of BCM+TOP yielded 0.02 ± 0.01 μg/mL of the plasma BCM concentration. No obvious morphological changes were observed in the Peyer’s patches and spleen (Figure 2B). No effects of BCM+TOP were observed on weights of the whole body, spleen, and intestine (Figure 2C–E). Examination of changes in subsets of intestinal mucosal lymphocytes showed an increase in CD20^+^ B cells and CD8^+^ T cells and a decrease in CD4^+^ T cells and Tregs (Figure 2F).

### 2.4. Effect of BCM on Colorectal Carcinogenesis

**Figure 3:**
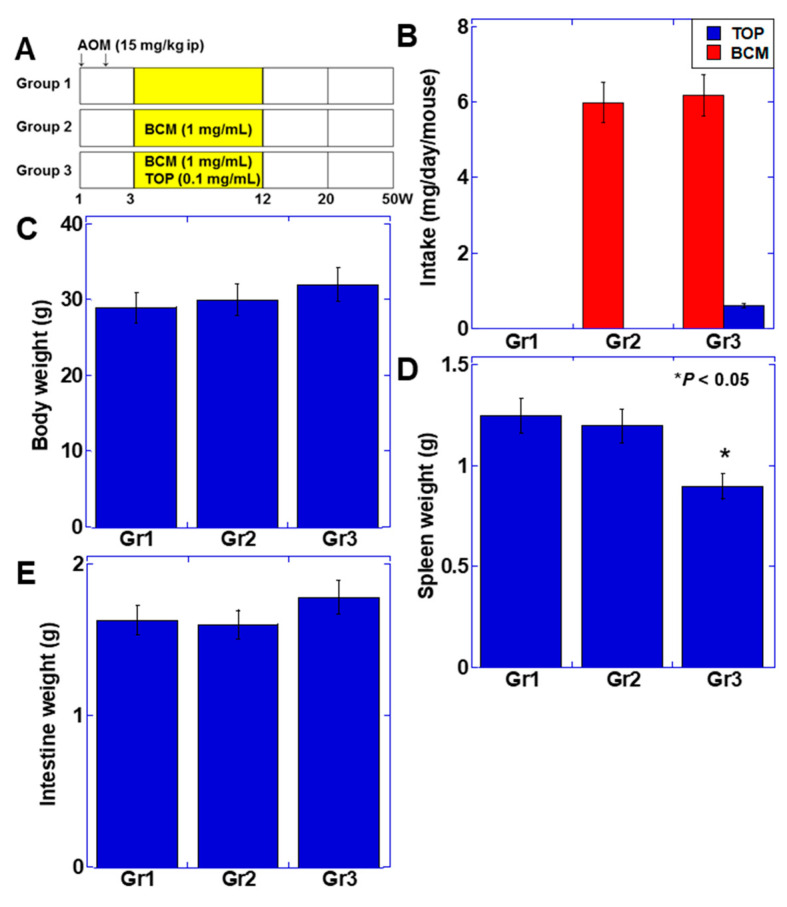
Effect of BCM on colon mouse carcinogenesis model. (**A**) AOM-injected BALB/c mice were treated with BCM+TOP mixed in drinking water. (**B**) Intake of BCM and TOP in each group. (**C**–**E**) Weights of the body, spleen, and intestine at week 50. Error bar, standard deviation from three independent trials. BCM, β-casomorphin-7; CD, cluster of differentiation; TOP, thiorphan; AOM, azoxymethane; Gr, group.

The above results suggest that BCM might promote intestinal immunity in a CD10-suppressed environment. Therefore, we examined the effect of BCM on colorectal carcinogenesis using a mouse azoxymethane carcinogenesis model (Figure 3). Azoxymethane (15 mg/kg body weight, ip) was administered twice, and BCM (1 mg/mL) and TOP (0.1 mg/mL) were mixed with drinking water and administered via ad libitum access to the water from week 3 to week 10 (Figure 3A). The administration of BCM+TOP yielded 0.02 ± 0.01 μg/mL of the plasma BCM concentration at week 12. Mice were euthanized at week 20 and 50 and assessed for aberrant crypt foci (ACF) and adenoma/adenocarcinoma, respectively. The average daily doses of BCM and TOP were 0.42 g/kg body weight and 0.042 g/kg body weight, respectively (Figure 3B). There were no clear differences in body weight and intestinal weight, but spleen weight decreased in the BCM+TOP group (group 3) (Figure 3C–E).

**Figure 4:**
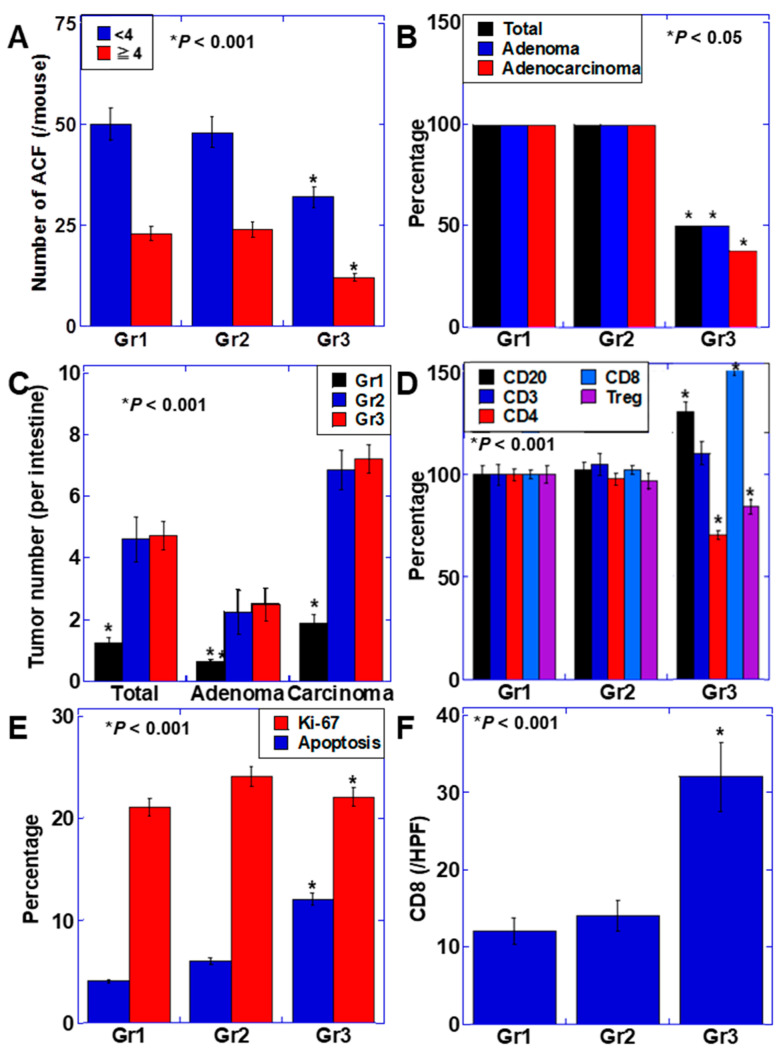
Effect of BCM on colon carcinogenesis in mice. (**A**) Number of ACF lesions in 10 mice at week 20. ACF was divided into clusters of <4 foci and ≥4 foci. (**B**) Percentage of tumor formation in 10 mice. (**C**) Number of adenoma and adenocarcinoma. (**D**) Lymphocyte subsets of the intestinal mucosa from which the tumor was removed. Differentiation markers were examined by ELISA. (**E**,**F**) Cell proliferation (Ki-67), apoptosis, and CD8^+^ T cells were examined by immunostaining. Error bar, standard deviation from three independent trials. BCM, β-casomorphin-7; ACF, aberrant crypt foci; TOP, thiorphan; Gr, group; ELISA, enzyme-linked immunosorbent assay; Treg, regulatory T cell.

The number of ACF at week 20 decreased in group 1 for both <4 clusters and ≥4 clusters (Figure 4A). Furthermore, the percentage of mice that developed adenomas and adenocarcinoma at week 50 was 100% in groups 1 (control) and 2 (BCM alone), while in group 3, the percentages were 50% for adenomas and 40% for adenocarcinomas (Figure 4B). The number of adenomas and adenocarcinomas in group 2 was similar to that in group 1 (control), while it was decreased in group 3 (BCM+TOP) (Figure 4C). Furthermore, no changes were observed in any of the lymphocyte subsets in the colonic mucosa in group 2 in comparison to the control. However, in group 3, CD4^+^ T cells and Tregs were decreased, whereas CD20^+^ cells, CD3^+^, and CD8^+^ T cells were increased compared to the other two groups (Figure 4D). Furthermore, when the proportions of proliferation (Ki-67), apoptosis (single strand-DNA, ss-DNA), and CD8^+^ T cell infiltration in adenocarcinoma were examined, there was no significant change in proliferation among any of the groups, but apoptosis and CD8^+^ T cell infiltration were increased in group 3 (BCM+TOP) in comparison with those in group 1 and 2 (Figure 4E,F).

From these results, it was considered that the combined use of BCM and TOP promoted colonic mucosal immunity and enhanced anti-tumor immunity, resulting in suppression of colon carcinogenesis.

### 2.5. Effect of BCM on Colorectal Cancer Metastasis

**Figure 5:**
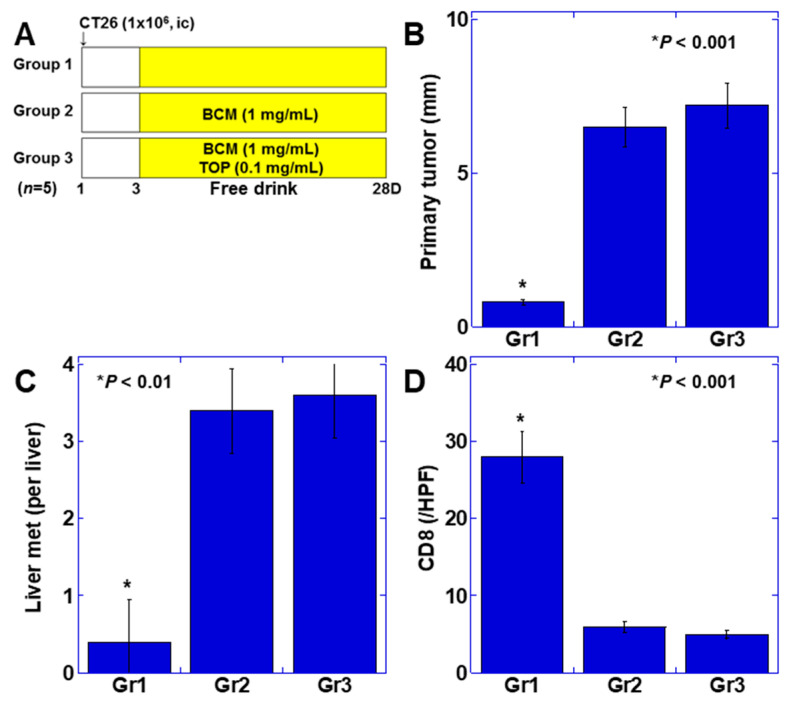
Effect of BCM on colon cancer metastasis in mice. (**A**) CT26 mouse colon cancer cells were inoculated in the cecum. The mice were treated with BCM+TOP mixed in drinking water. (**B**) Diameters of primary tumors in the cecum. (**C**) Number of liver metastasis. (**D**) CD8^+^ T cells in the primary tumors were examined by immunostaining. Error bar, standard deviation from three independent trials. BCM, β-casomorphin-7; TOP, thiorphan; Gr, group.

Finally, we examined the effect of BCM on liver metastasis of colorectal cancer. A mouse cecal orthotopic colon cancer model, in which it is easy to evaluate effects of the immune environment on the mucosa, was developed using BALB/c mice and the syngeneic CT26 mouse colon cancer cells (Figure 5A). In the BCM alone group (group 2), the diameter of the primary tumors of the cecum at 4 weeks after inoculation was similar to that observed in the control (group 3). In contrast, the tumor diameter in the BCM+TOP group (group 1) was approximately 11% of that of the control group (Figure 5B). The number of liver metastases was not different between groups 2 and 3, but no metastases were found in group 1 (Figure 5C). Intratumoral infiltrated CD8^+^ T cells in the primary lesion increased approximately five-fold in group 1 compared to that in groups 2 and 3 (Figure 5D).

Thus, it was considered that liver metastasis of colorectal cancer was suppressed by the promotion of anti-tumor immunity in colorectal mucosa through the combined use of BCM and TOP.

## 3. Discussion

In the present study, BCM was demonstrated to be degraded and inactivated by CD10, which is present in the brush border of the intestinal mucosa. When CD10 was suppressed by its inhibitor TOP, BCM treatment increased CD8^+^ T cells and decreased CD4^+^ cells and Tregs in the mucosa. As a result, both colorectal carcinogenesis and colorectal cancer liver metastasis were suppressed.

Our data showed that BCM has an effect on lymphocytes. BCM is known to bind and activate the μ-opioid receptor (MOR) [22,23], and affect the nerve, digestive, and immune functions that involve MOR [24]. Regarding the action of MOR on lymphocytes, suppression of opioid receptors by naloxane treatment was reported to increase CD4^+^ T cells and Tregs and decrease CD8^+^ T cells and Th-17 cells in spleen lymphocytes [25]. This finding is in accordance with our data, suggesting that the effect of BCM on lymphocytes is mediated by opioid receptors. In addition, it has been reported that BCM promotes the proliferation of peripheral blood immune cells and the secretion of interleukin (IL)-4, IL-13, and interferon (IFN)-γ [26]. In CD8^+^ T cells expressing MOR and delta-opioid receptor, their ligand, methionine-enkephalin (MENK) causes cell proliferation, activation, increased cytotoxic activity against tumor cells, and increased IFNγ secretion, inducing cytotoxic lymphocyte activity [27,28].

BCM acts as a 5-HT-2-serotonin receptor antagonist and is involved in the regulation of the serotonergic system [29]. Activation of 5-HT2-serotonin receptor increases CD4^+^ T cells and Tregs and decreases Th1/Th17 cytokine production [30,31,32]. Conversely, serotonin relatively reduces the frequency of CD8^+^ T cells in intestinal mucosal lymphocytes [33]. The inhibitory effect of BCM on serotonin receptor is consistent with the effect of BCM on Tregs and CD8^+^ T cells. In addition, BCM is known to have a histamine-releasing effect [12]. Histamine suppresses Tregs and CD4^+^ T cells [34,35] and promotes recruitment and activation of CD8^+^ T cells [36]. Thus, BCM is thought to affect lymphocytes through multiple mechanisms, such as MOR activation, 5-HT2-serotonin receptor inhibition, and histamine secretion promotion. Indeed, our carcinogenesis model, phosphorylated PLCγ1, which is downstream of 5-HT2-serotonin receptor, was decreased, whereas histamine was increased in the intestine and spleen.

The degradation system is considered to play an important role in the activity of BCM. Dipeptidyl peptidase IV (DPPIV; EC 3.4.14.5) is the major degrading enzyme of BCM [24,37]. In peripheral blood mononuclear cells of patients with atopic dermatitis, BCM promotes MOR expression and reduces DPPIV expression, leading to exacerbation of the allergy. Thus, suppression of BCM degradation amplifies the BCM effect. In our data, the BCM+TOP model (Figure 2) showed very low plasma BCM levels and did not change lymphocyte subsets (data not shown). It was considered that this was because plasma BMP was degraded by DPP-4 expressed in vascular endothelial cells.

The CD10 that we examined in this study has various alternative names, including membrane metalloendopeptidase, common acute lymphocytic leukemia antigen, neprilysin, neutral endopeptidase, enkephalinase, and atriopeptidase [NCBI, OMIM]. CD10 is known to degrade short peptides such as natriuretic peptides, angiotensin II, bradykinin, apelins, substance P, and adrenomedullin [38]. CD10 expression is widespread in the intestinal mucosa [39]. Incubation of BCM with brush border of the intestinal mucosa degrades 95% of BCM in 24 h [20].

In our study, a short fragment of the N-terminal tyr-pro of iodine-labeled BCM was cleaved by CD10 and the intact BCM disappeared after 24 h. From these facts, it is considered that CD10 degrades BCM in the same manner as DPPIV. In addition, the degraded fragment of BCM (BCM3-7) after cleavage of the N-terminal tyr-pro lost its action on lymphocytes. Thus, it is considered that most of the BCM produced from milk is degraded by CD10 expressed on the intestinal mucosa and thereby BCM activity is lost. Therefore, it is necessary to inhibit the activity of CD10 in order to retain the activity of orally administered BCM on lymphocytes in vivo. In our experiments, it was shown that co-administration of TOP with BCM allowed BCM to exert its effect on lymphocytes.

Our data show that BCM, a milk degradation product, is degraded in the intestinal tract and loses its effectiveness under physiological conditions; however, suppressing BCM degradation with a CD10 inhibitor lowered the suppressive system of intestinal mucosal immunity and increased CD8^+^ T cells. The result was suppression of colorectal carcinogenesis and cancer metastasis. These results are important because they indicate that inexpensive food nutrition might suppress the development and progression of colorectal cancer.

In the present study, administration of BCM with CD10-suppressing TOP increased the infiltration of CD8^+^ T cells in the primary lesion and suppressed liver metastasis. In the present study, we have not examined blood BCM concentration or infiltration of CD8^+^ T cells in metastatic lesions. It is considered that DPPIV expressed in endothelial cells degrades blood BCM. However, there are few findings regarding blood BCM under the condition of CD10 inhibition. Therefore, future studies should examine the effect of CD10 inhibitors on blood BCM. As BCM is a substance produced by ingesting cow milk, it is also necessary to examine its effect on mucosal immunity using a CD10 inhibitor in combination with milk ingestion. The effect of BCM on mucosal immunity is also considered to be similar to the effect in diseases due to abnormal immunity activation, such as in ulcerative colitis; in the future, it is necessary to study such diseases.

In conclusion, our findings suggest that BCM might be a novel and effective adjuvant to cancer immunotherapy. So far, no clinical trials have been conducted on cancers using BCM. Future clinical studies are required to validate our findings.

## 4. Materials and Methods

### 4.1. Cell Culture

The CT26 mouse colon cancer cell line was a kind gift from Professor I. J. Fidler (MD Anderson Cancer Center, Houston, TX, USA). CT26 cells were cultured in Dulbecco’s modified Eagle’s medium (Wako Pure Chemical Industries, Ltd., Osaka, Japan) supplemented with 10% fetal bovine serum (Sigma-Aldrich Chemical Co., St. Louis, MO, USA).

### 4.2. Animals

Five-week old male BALB/c mice were purchased from SLC Japan (Shizuoka, Japan). The animals were maintained in a pathogen-free animal facility under 23 °C, 50% humidity, and a 12-h light/12-h dark cycle environment. The animal study was conducted in accordance with the institutional guidelines approved by the Committee for Animal Experimentation of Nara Medical University, Kashihara, Japan, following current regulations and standards of the Japanese Ministry of Health, Labor and Welfare (approval nos. 9559, 11365, 11528, 11569). Animals were acclimated to their housing for seven days before the start of the experiment. Mice were fed with a CE-2 standard diet (CLEA Japan, Inc., Tokyo, Japan).

### 4.3. Spleen Cells

Under anesthesia with 3% sevoflurane inhalation (Wako Pharmaceuticals Inc., Osaka, Japan),

Spleens were excised aseptically from 5-week-old male BALB/c mice, minced and pressed through 23 gauze needle into regular medium in 10 cm dishes. After overnight culture, floating cells were collected as spleen cells.

### 4.4. Mouse Non-Cancer Model

BALB/c mice (male, 5-week-old) were administered with BCM (10 mg/kg body weight, in gavage) and thiorphan (TOP, 1 mg/kg body weight, intraperitoneal injection) once a day. After 5 days of treatment, the mice were euthanized.

### 4.5. Mouse Colon Carcinogenesis Model

BALB/c mice (male, 5-week-old, 20 mice per group) were injected azoxymethane (AOM; 15 mg/kg body weight) intraperitoneally twice (week 1 and 2). BCM (1 mg/mL in water) + TOP (0.1 mg/mL; group 1) or BCM (1 mg/mL in water; group 2) or water alone (group 3; control) were administered via free drinking water from week 3 to week 11. At week 20, 10 mice were euthanized to assess formation of ACF. At week 50, another 10 mice were euthanized to assess tumor formation. Weights of body, spleen, and large intestine were measured at euthanasia.

For assessing ACF, the colon was removed, flushed with normal saline, opened from the cecum to anus, and fixed flat on the plastic plate in 10% buffered formalin. The colon was stained with methylene blue and observed by a stereomicroscope (Nikon, Tokyo, Japan) for counting ACF; clusters < 4 foci and clusters ≥ 4 foci were distinguished.

For assessing colon tumors, the colon was removed, flushed with normal saline, and opened from the cecum to anus. The observed elevated neoplastic lesions were removed and fixed with 10% buffered formalin for histological analysis. In tumors at week 50, adenoma and adenocarcinoma were distinguished.

For analysis of mucosal protein levels, the colon mucosa was scraped in each group, frozen under liquid nitrogen, and stored at −80 °C until further analysis.

### 4.6. Orthotopic Liver Metastasis Model

For the establishment of liver metastasis models, CT26 cancer cells (1 × 10^6^ in 40 μL phosphate-buffered saline) were inoculated into the cecal submucosal layer of a syngeneic BALB/c mouse. Mice were euthanized at 4 weeks after inoculation to assess liver metastasis. The excised livers were sectioned into 2-mm-thick slices, and metastatic foci were counted using a stereomicroscope (Nikon) [40].

### 4.7. Histological Analysis

The formalin-fixed tissues were dehydrated and embedded in paraffin. After slicing the created block to 3 μm, hematoxylin and eosin staining was performed to observe the morphology.

### 4.8. Immunohistochemistry

Consecutive 4-μm sections were immunohistochemically stained using the immunoperoxidase technique described previously [41], with primary antibodies for Ki-67 (proliferation, DAKO, Glostrup, Denmark), single strand DNA (ss-DNA, Medical and Biological Laboratories, Nagoya, Japan), and mouse CD8 (Abcum, Cambridge, UK). Thereafter, they were treated with appropriate secondary antibodies (Medical and Biological Laboratories) (all 0.2 μg/mL). The tissue sections were then color-developed with diamine benzidine hydrochloride (DAKO, Glostrup, Denmark), and counterstained with Meyer’s hematoxylin (Sigma). Proliferation and apoptosis were examined by observation of 1000 nuclei for positive antibody staining. CD8^+^ T cells were counted in 20 high power fields.

### 4.9. Protein Extraction

To prepare whole cell lysates, cells were washed twice with cold PBS and harvested. Colon mucosa and spleen tissues were washed with cold PBS and pelleted with a sonicator (QSONICA, WakenBtech Co. Ltd., Kyoto, Japan). Cells or tissues were lysed with 0.1% SDS-added RIPA buffer (Thermo Fisher). Protein assays were performed using a Protein Assay Rapid Kit (Wako).

### 4.10. Enzyme-Linked Immunosorbent Assay (ELISA)

ELISA kits were used for measuring protein levels of CD20 (Proteintech Group, Inc., Rosemont, IL, USA), CD3 (Novus Biologicals, LLC, Centennial, CO, USA), CD4 (RayBiotech Life, Inc., Peachtree Corners, GA, USA), CD8 (Abcam, Cambridge, MA, USA), Treg (FoxP3, LSBio, Seattle, WA, USA), BCM (Creative Diagnostics, Shirley, NY, USA), phosphorylated phospholipase Cγ1 (pTyr771), and histamine (Assaybiotechnology, Fremont, CA, USA). The assay was performed according to the manufacturer’s instructions, and whole cell lysates were used for the measurements.

### 4.11. Degradation of BCM by CD10

BCM was purchased from Sigma. Di-amino acid peptide (tyr-pro; 1st and 2nd amino acids of BCM) was synthesized by Peptide Institute Inc. (Ibaraki, Japan). BCM and the tyr-pro peptide were labeled by chloramine-T method with ^125^I (J-RAM, Tokyo, Japan) using Pierce™ iodination beads (Thermo Fisher). Labeled BCM was incubated with CD10 in neutral peptidase assay buffer (40 mM Tris-HCl pH 7.1, 4 mM MgCl_2_, 0.5 mM ZnCl_2_) for 16 h. Thereafter, the solution was incubated with anti-BCM antibody (Abnova, Taipei City, Taiwan) at 37 °C for 2 h. The solution was subjected to immunoblot analysis in 20–25% sodium dodecyl sulfate-polyacrylamide gradient gels (Multigel II, Cosmo Bio, Tokyo, Japan). The radioactivity of the solution was also measured by a fluid scintillation counter (LSC-8000, Hitachi, Tokyo, Japan).

### 4.12. Statistical Analysis

Statistical significance was calculated using a two-tailed Fisher’s exact test, an ordinary analysis of variance, and InStat software (GraphPad, Los Angeles, CA, USA). Correlations were tested using Pearson’s correlation test. A two-sided *p* value of <0.05 was considered to indicate statistical significance.

## Figures and Tables

**Table 1:** Protein levels of phosphorylated phospholipase C (PLC) γ1 and histamine.

Protein ^1^		Control	BCM
Phosphorylated PLCγ1	Spleen cells	100 ± 5 ^2^	71 ± 5
Histamine	Medium	100 ± 3	138 ± 6

^1^ Protein levels were examined by ELISA. ^2^ Protein levels were represented as percentage. Control is set as 100.

## Data Availability

Not applicable.

## Abbreviation

BCM	β-Casomorphin-7
TOP	thiorphan
CD	cluster of differentiation
ACF	aberrant crypt foci
ss-DNA	single strand-DNA
MOR	μ-opioid receptor
IL	interleukin
Treg	regulatory T cell
MENK	methionine-enkephalin
HT	hydroxytryptamine
Th	T cell helper
DPP	dipeptidyl peptidase
